# The Relationship Between Proactive Behavior and Work-Family Conflict: A Moderated Mediation Model

**DOI:** 10.3389/fpsyg.2021.657863

**Published:** 2021-05-03

**Authors:** Zilong Cui, Yuyin Li

**Affiliations:** ^1^Department of Human Resource Management, Yatai College of Business Administration, Jilin University of Finance and Economics, Changchun, China; ^2^Department of Public Service Management, College of Economics and Management, Dali University, Dali, China; ^3^Department of Public Relations, College of Economics and Management, Dali University, Dali, China

**Keywords:** proactive behavior, workplace anxiety, work-family conflict, perspective taking, emotional intelligence

## Abstract

This study aimed to explore the linking mechanisms and conditional processes underlying the relationship between proactive behavior and work-family conflict. Considering the conservation of resources theory, we argue that workplace anxiety mediates the relationship between proactive behavior and work-family conflict. Furthermore, we suggest that immediate supervisor perspective taking and employee emotional intelligence moderate this proposed indirect effect. Two-wave, multisource lagged data were collected from 450 employees of seven domestic Chinese firms to examine the hypothesized moderated mediation model. Our findings support the hypothesis that proactive behavior is positively related to work-family conflict and that workplace anxiety partially mediates this relationship. Immediate supervisor perspective taking moderates the positive association of proactive behavior with workplace anxiety and the indirect relationship between proactive behavior and work-family conflict through workplace anxiety. Emotional intelligence moderates the positive association of proactive behavior with workplace anxiety and the indirect relationship between proactive behavior and work-family conflict through workplace anxiety. The results deepen our theoretical understanding of the consequences of proactivity by demonstrating the positive associations between proactive behavior and work-family conflict. The current study also contributes to the literature by identifying workplace anxiety as a mediating mechanism explaining the relationship between proactivity and work-family conflict. Furthermore, supervisor perspective taking and employee emotional intelligence moderate the above mediating effect.

## Introduction

China has experienced rapid economic growth over the past 30 years (Rasool, 2021). China has become the global leader in manufacturing operations and the second largest economic power in the world (Li, 2018). The 2019 report from China’s National Bureau of Statistics showed that China’s manufacturing and high-tech manufacturing industries, IT services, and financial sectors grew by 6.0, 8.8, 18.7, and 8.7%, respectively. Chinese companies have also developed rapidly, and some of them have become world-renowned companies (e.g., Huawei, Alibaba, China Construction Bank, and China FAW Group). However, with the globalization of the economy and the rapid development of technology, the context in which organizations find themselves has changed dramatically. Thus, if organizations want to gain an advantage in fierce competition, it is no longer sufficient for employees to complete their tasks in line with their job descriptions. Rather, enterprises need employees to be self-starters and use their initiative to scan the workplace environment in order to identify opportunities and threats, anticipate and act on future problems, plan in anticipation of such problems, and take the initiative to implement ideas (Campbell, 2000; Crant, 2000; Fay and Frese, 2001; Griffin et al., 2007). In the organizational context, these behaviors are included within the concept of proactive behavior, whereby an employee engages in self-directed and future-oriented changes in actions in his or her work environment or work role (Griffin et al., 2007). Proactivity has received much attention in recent years because it benefits organizations in many ways, such as by increasing organizational effectiveness (Griffin et al., 2007; Grant et al., 2010) or enhancing long-term working conditions (Frese et al., 2007). Nonetheless, a small but growing body of research has revealed that there is a dark side to proactive behavior as it can have a negative impact on an individual’s health, well-being, and family life (Fay and Huttges, 2017; Cangiano et al., 2019; Altura et al., 2020; Zhang et al., 2020). Work-family conflict refers to role pressure from one domain (work and family) transferring to another domain (family and work; Kahn et al., 1964; Greenhaus and Beutell, 1985). Few previous studies have addressed proactive behavior that may spill over into employees’ family lives and lead to work-family conflict (Bolino and Turnley, 2005; Lin and Yu, 2019; Zito et al., 2019; Altura et al., 2020). However, the theoretical understanding of the mechanism and processes between proactive behavior and work-family conflict is far from complete. To fill this research gap, we aim to explore the mechanisms and processes underlying the effect of proactive behavior on work-family conflict by including employees from seven enterprises (one banking enterprise, three enterprises in the high-technology industry, two enterprises in the manufacturing industry, and one trading company) in Northeast China as research participants.

Specifically, proactive behavior is regarded as a self-regulation process that involves envisioning, planning, enacting, and reflecting (Grant and Ashford, 2008). Based on the conservation of resources (COR) theory (Hobfoll, 1989) and the work-home resource (W-HR) model (ten Brummelhuis and Bakker, 2012), we theorize that proactive behavior consumes personal resources that may impede the work-family interface and cause work-family conflict. Because workplace anxiety denotes an emotional state characterized by nervousness, uneasiness, and tension associated with resource loss (Buchwald, 2010; Cheng and McCarthy, 2018; Samma et al., 2020), we suppose that proactive behavior is positively related to workplace anxiety, which in turn increases work-family conflict. Moreover, we hypothesize that immediate supervisor perspective taking and employee emotional intelligence (EI) attenuate the positive effect of proactive behavior on work-family conflict *via* workplace anxiety. Perspective taking has been characterized as a process in which individuals try to imagine or understand others’ viewpoints (Galinsky et al., 2005). EI refers to the ability to produce, acknowledge, express, understand, and assess one’s own and others’ emotions and successfully cope with environmental demands and stresses (Van Rooy and Viswesvaran, 2004). Based on the COR theory (Hobfoll, 1989) and the W-HR model (ten Brummelhuis and Bakker, 2012), supervisors’ perspective taking and employees’ EI are contextual resources, and personal resources help individuals prevent work-family conflicts caused by resource loss. In summary, we introduce a moderated mediation model in which workplace anxiety serves as a mediator of the effect of proactive behavior on work-family conflict. Immediate supervisor perspective taking moderates the positive indirect relationship of employee proactive behavior with employee work-family conflict through workplace anxiety such that the relationship is less positive when the employee’s immediate supervisor’s perspective taking is high; in addition, employee EI moderates the positive indirect relationship of proactive behavior with work-family conflict through workplace anxiety such that the relationship is less positive when employee EI is high.

**Figure 1:**
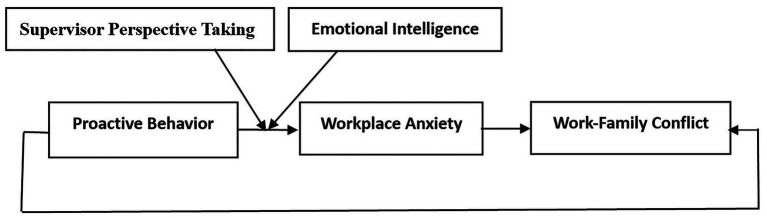
Research model.

This research topic is important because if proactive behavior benefits only the organization and an employee’s career but harms the employee’s family life, then the sustainability of this behavior is questionable. Accordingly, our study seeks to make several theoretical contributions to the literature. Firstly, our study answers the call for a more empirical investigation of the outcomes of proactive behavior in organizations by exploring the relationship between proactive behavior and work-family conflict. Secondly, although recent studies have focused on how proactive behavior influences work-family conflict, relatively little is known about the underlying mechanism of their relationship. By integrating the COR theory and the W-HR model, our research contributes to explaining the relationship between proactive behavior and work-family conflict. Thirdly, by identifying and examining supervisors’ perspective taking and employees’ EI as moderators of proactive behavior, this study further enriches the boundary conditions for the proactivity theory. We summarize the above analysis in the theoretical model shown in Figure 1.

The structure of the paper is as follows. In second section, we provide a Literature Review. Third section describes the Theoretical Framework and Hypotheses Development. Fourth section presents the Material and Methods, and fifth section explains the Results. Sixth section provides the Discussion, and seventh section Conclusion. Eighth section presents the Practical Implications. Finally, the final section of this paper describes the limitations of the study and future research.

## Literature Review

### Proactive Behavior

Proactive behavior is defined as an employee engaging in self-directed and future-oriented changes in action in his or her work environment or work role (Fay and Frese, 2001; Grant and Ashford, 2008; Parker et al., 2010). This definition indicates three characteristics of proactive behavior: self-initiation, future focus, and change orientation. Grant and Ashford (2008) suggested that proactive behavior is regarded as a self-regulation process that involves envisioning, planning, enacting, and reflecting. Thus, when people are proactive at work, they independently think, deliberate, plan, calculate, and act in advance (Grant and Ashford, 2008). Previous research has confirmed that individual and situational factors jointly shape individuals’ proactive behavior (Bateman and Crant, 1993; Parker et al., 2006; Lebel, 2017; Wu and Parker, 2017; Binyamin and Brender-Ilan, 2018; Cai et al., 2019). Existing research has also highlighted that various forms of proactivity have positive effects on outcomes such as work performance and career success (Griffin et al., 2007; Grant et al., 2010). Compared to the antecedents of proactive behavior, the consequences of proactive behavior are not well understood (Liu et al., 2019).

### Work-Family Conflict

Work-family conflict occurs when role pressure from one domain (work and family) transfers to another domain (family and work; Kahn et al., 1964; Greenhaus and Beutell, 1985). Following the perspective of Greenhaus and Beutell (1985), time-based conflict, strain-based conflict, and behavior conflict are three sources of work-family conflict. Specifically, work behavior causes work-family conflict because it is incompatible with family role expectations (Greenhaus and Beutell, 1985). For instance, a family role requires an individual to be warm, nurturing, and empathetic. Such expectations are in contrast to job role expectations, including requirements to be objective, neutral, and calm.

### Workplace Anxiety

Workplace anxiety refers to an emotional state reflecting nervousness, uneasiness, and tension in the workplace (Cheng and McCarthy, 2018). When individuals are upset by things such as meeting timelines, uncertainty regarding things that may or may not happen, and social conflict, they may more easily experience anxiety (Miceli and Castelfranchi, 2005; MacLeod and Mathews, 2012; Grupe and Nitschke, 2013; Cheng and McCarthy, 2018; Rasool et al., 2020). Previous literature has suggested that workplace anxiety is positively associated with unethical workplace behavior, risk-taking behavior, and organizational turnover (Rodell and Judge, 2009; Kouchaki and Desai, 2015; Mannor et al., 2016).

### Perspective Taking

Perspective taking has been conceptualized as a process in which an observer attempts to infer the thoughts, motives, and/or feelings of others (Galinsky et al., 2005; Parker et al., 2008). Perspective taking is often considered to be an active cognitive process – perspective takers mentally simulate what it would be like to be someone else and to see the world from that person’s perspective. Perspective taking has many positive benefits for organizations because it can increase psychological closeness, coordination, cooperation, proactive service performance, and helping (Falk and Johnson, 1977; Galinsky et al., 2005; Shih et al., 2009; Ku et al., 2015; Huo et al., 2019).

### Emotional Intelligence

EI has been defined as the disposition or ability allowing one to develop, identify, express, understand, and appraise one’s own and other people’s emotions to successfully cope with environmental demands and pressures (Van Rooy and Viswesvaran, 2004). George (2000) and Mayer et al. (1999) suggested that EI involves four aspects: (a) the perception of emotions, (b) the integration and assimilation of emotions, (c) the understanding of emotions, and (d) emotion regulation. The perception of emotions refers to individuals’ ability to accurately identify emotions in themselves and in others. The integration and assimilation of emotions allow people to use emotions to guide their thinking and facilitate decision making. The understanding of emotions concerns how people understand their emotions. The fourth dimension, emotion regulation, refers to the extent to which individuals can manage their own and others’ emotions. EI has been regarded as a predictor of various domains, such as job performance, leadership, emotional labor, trust, work-family conflict, stress, mental health, and well-being (Jordan et al., 2002; Fulmer and Barry, 2004; Daus and Ashkanasy, 2005; Humphrey et al., 2008; Sánchez-Álvarez et al., 2016).

## Theoretical Framework and Hypothesis Development

### Proactive Behavior and Work-Family Conflict

According to Bindl et al. (2012), proactive behavior is a self-regulation progress that involves envisioning, planning, enacting, and reflecting. Thus, proactivity requires thinking independently, carefully deliberating, planning, and calculating, and acting in advance (Grant and Ashford, 2008). Regarding role theory, when people devote more time and energy to their work roles, they tend to have less time and energy to spend with family members and fulfill their family duties (Arlie, 1997). Thus, proactive employees are more inclined to prioritize work demands and focus less attention on family demands and obligations (Altura et al., 2020). Consequently, proactive employees are likely to experience conflict between work and family because they do not sufficiently fulfill family obligations. Indeed, previous studies have suggested a positive relationship between proactive behavior and work-family conflict. For example, Altura et al. (2020) reported that proactive individuals are more inclined to prioritize their work needs, resulting in greater work to family interference. Bolino and Turnley (2005) suggested that higher levels of individual initiative are associated with higher levels of work-family conflict. Zito et al. (2019) argued that job crafting, which is considered a specific proactive behavior, is positively correlated with work-family conflict. Taken together, the above arguments and evidence suggest the following:

*Hypothesis 1*: Proactive behavior is positively related to work-family conflict.

### Proactive Behavior and Workplace Anxiety

Proactivity may require an employee to focus on the future, challenge the status quo, and “mak[e] things happen” (Parker et al., 2019). Although proactive behavior may promote career success and performance for employees (Fuller and Marler, 2009; Thomas et al., 2010; Tornau and Frese, 2015), scholars have argued that proactive behavior is not always appreciated by leaders and coworkers and may lead to some negative outcomes (Spychala and Sonnentag, 2011; Fay and Huttges, 2017; Pingel et al., 2019; Cangiano et al., 2020; Zhang et al., 2020). In this paper, we argue that proactivity generates workplace anxiety. The reasons are as follows. Firstly, proactive work behavior involves a future focus. Being proactive often results in changes to established work processes, and the result of being proactive is always unknown (Bolino et al., 2010). This may lead proactive individuals to experience uncertainty and unpredictability at work. Secondly, the changing nature of proactive behavior may result in changes to work roles, organizational norms, and work procedures (Fay and Frese, 2001; Grant and Ashford, 2008). In an interdependent work environment, violating or changing the prescribed pattern means that proactivity will affect other people’s work. Thus, being proactive is not always welcome or may even be rejected or envied by others (Bolino et al., 2010; Sun et al., 2020; Zhang et al., 2020). For example, previous studies have suggested that, although proactive behavior is necessary, it should conform to the leader’s expectations (Campbell, 2000; Grant et al., 2010). For this reason, engaging in proactive behavior is considered “risky” because it generates social friction among proactive employees. Fay and Huttges (2017) proposed that these aversive experiences of social friction and social tension negatively affect individuals. Furthermore, when people are proactive at work, they carefully deliberate, plan, calculate, and act in advance (Grant and Ashford, 2008). Their proactive behavior consumes resources in terms of time and energy (Strauss et al., 2017; Pingel et al., 2019; Urbach and Weigelt, 2019). Because time and energy are limited resources, proactive employees also often experience time conflict and fatigue at work (Fay and Frese, 2001; Cangiano et al., 2020). Therefore, we can expect that the uncertainty, time conflict, and social friction caused by proactive behavior will lead proactive employees to experience workplace anxiety. Based on this rationale, we hypothesize the following:

*Hypothesis 2*: Proactive behavior is positively related to workplace anxiety.

### Workplace Anxiety and Work-Family Conflict

We expect that a positive relationship exists between workplace anxiety and work-family conflict. Anxiety is a symptom of strain that makes individuals feel nervous, uneasy, and tense (McCarthy et al., 2016). It has been suggested that symptoms of strain, such as tension, anxiety, and fatigue in one’s role (work or family), can affect one’s performance in another role (family or work; Sell and Schuler, 1981; Michel et al., 2011). Thus, the negative affect of an anxious employee is more likely to spill over into the family domain and interfere with the performance of family duties. Previous literature has shown that anxious employees are more inclined than employees who are not anxious to interact with their partners with less warmth and supportiveness (Salovey and Rosenhan, 1989; Matthews et al., 1996) and express more criticism and dissent (Story and Repetti, 2006) when they return home from work. Furthermore, anxious employees experience cognitive interference, which refers to the tendency to spend considerable time ruminating on task-irrelevant or off-task thoughts (Sarason et al., 1996). For example, when employees feel anxious about a work task or worry about expected failure, these feelings will occupy their minds and cause them to spend a large amount of time cognitively processing and thinking about these tasks (MacIntyre and Gardner, 1994; Cheng and McCarthy, 2018). Therefore, interference with mental processes due to workplace anxiety can be expected to inevitably reduce performance in the family domain. Michel et al. (2011) performed a meta-analysis that showed that negative affective personal traits characterized by stress, anxiety, and dissatisfaction had a significant positive effect on work-family conflict. According to the COR theory (Hobfoll, 1989), workplace anxiety triggers an emotion-focused coping strategy that causes a resource drain, which manifests in the depletion of emotion regulation. Emotional exhaustion, in turn, increases work-family conflict (Grandey, 2003; Goldberg and Grandey, 2007; Trougakos et al., 2020). Based on the proposition above, we hypothesize the following:

*Hypothesis 3*: Workplace anxiety is positively related to work-family conflict.

### The Mediating Role of Workplace Anxiety

We expect proactive behavior to have indirect effects on work-family conflict through workplace anxiety. That is, individuals who engage in proactive behavior at work experience workplace anxiety, which leads to work-family conflict. Considering the basic assumptions of the COR theory (Hobfoll, 1989) and the W-HR model (ten Brummelhuis and Bakker, 2012), initial resource losses will lead to future resource losses. As such, work resource loss primarily leads to stress and strain and further results in poorer outcomes at home (Halbesleben et al., 2014). Thus, proactive behavior at work requires high levels of personal resource consumption (e.g., time, energy, and self-effort), and the depletion of personal resources caused by proactivity leads to job strain and anxiety (Bolino et al., 2010; Hahn et al., 2012; Cangiano et al., 2019; Pingel et al., 2019). In turn, workplace anxiety is more likely to spill over to the home domain and cause work-family conflict (Doby and Caplan, 1995). Based on the proposition above, we hypothesize the following:

*Hypothesis 4*: Workplace anxiety mediates the relationship between proactive behavior and work-family conflict.

### The Moderating Role of Perspective Taking

Leaders’ perspective taking can be transformational if they are able to assume the perspective of a follower (Parker et al., 2008; Gregory et al., 2011). We expect an immediate supervisor’s perspective taking to moderate the relationship between employee proactive behavior and workplace anxiety. Firstly, perspective taking serves to increase openness to diverse perspectives, increases the desire to help, builds trust, and facilitates knowledge-sharing behavior (De Dreu et al., 2000; Galinsky and Moskowitz, 2000; Moates et al., 2007; Flinchbaugh et al., 2016). Proactive employees whose leaders score high in perspective taking may be more likely to obtain help from their leaders. With help from their leaders, employees can successfully implement proactive behavior with less time, energy, and effort, which ultimately relieves their workplace anxiety. Furthermore, leaders’ perspective taking helps to build trust with subordinates. When proactive employees feel trusted, risky proactive behavior is perceived as safe, with reduced uncertainty and social tension. According to the self-determination theory (Ryan and Deci, 2008), trusted employees who feel competent tend to report feeling greater vigor and less anxiety (Spreitzer, 1995; Thatcher et al., 2007). Thus, when an immediate supervisor is strong in perspective-taking ability, proactive employees are more likely to be motivated (Galinsky and Schweitzer, 2016). Conversely, when an immediate supervisor is weak in perspective-taking ability or is even punitive, proactive employees report experiencing more workplace anxiety (Cangiano et al., 2019).

Moreover, we argue that the positive effect of proactivity on work-family conflict through workplace anxiety may be weaker for employees who experience high levels of perspective taking from their supervisors. Specifically, we expect immediate supervisor perspective taking to moderate the indirect effects of employee proactivity on employee work-family conflict. Evidence suggests that leaders’ openness to proactivity can facilitate the positive effect of proactivity (Huo et al., 2013; Parker et al., 2019). Integrating the COR theory (Hobfoll, 1989) and the W-HR model (ten Brummelhuis and Bakker, 2012), we expect that leaders’ perspective taking, which represents their support, facilitates employees’ personal resource transitions from the work domain to the home domain. Thus, proactive employees whose leaders score high in perspective taking tend to experience lower anxiety and less work-family conflict. In contrast, when the supervisor exhibits low levels of perspective taking, proactivity among employees is more likely to trigger workplace anxiety and further cause work-family conflict. In summary, we hypothesize the following:

*Hypothesis 5*: Immediate supervisor perspective taking moderates the positive relationship between proactive behavior and workplace anxiety so that the relationship is weaker for immediate supervisor with high levels of perspective taking (in contrast to immediate supervisor with low levels of perspective taking).

*Hypothesis 6*: Immediate supervisor perspective taking moderates the positive impact of proactive behavior on work-family conflict *via* workplace anxiety so that the relationship is weaker for immediate supervisor with high levels of perspective taking (in contrast to immediate supervisor with low levels of perspective taking).

### The Moderating Role of Emotional Intelligence

We expect EI to moderate the relationship between proactive behavior and workplace anxiety such that the relationship is less positive when EI is high than when EI is low. People with higher EI have the ability to better perceive, understand, and manage their emotions. Therefore, people with high EI typically have a more positive mood and are able to cope with negative emotional states associated with mood and anxiety (Schutte et al., 2002; Foster et al., 2018; Martínez-Monteagudo et al., 2019). In previous studies, EI has been found to be negatively related to social interaction anxiety, writing anxiety, communicative anxiety, and foreign language anxiety (Summerfeldt et al., 2006; Dewaele et al., 2008; Huerta et al., 2017). Therefore, although proactive behavior triggers workplace anxiety, EI helps employees alleviate this negative emotional state. Prior studies have found that, because people with EI are able to regulate their moods, EI buffers the negative effects of stress (Görgens-Ekermans and Brand, 2012; Fu et al., 2020). Moreover, people with EI can perceive and manage others’ emotions. Thus, people with high EI take into account the feelings of others at work. Hence, high EI can help people reduce workplace anxiety caused by interpersonal tension from their proactive behavior. In a similar vein, Grant (2013) proposed that knowledge of emotion regulation enables employee voice to elicit favorable performance evaluations and, thus, reduces the threatening interpersonal aspects of employee voice. Hence, EI reduces the positive associations between proactive behavior and workplace anxiety. In contrast, for proactive employees who have low levels of EI or relational knowledge, their proactivity is not accepted or appreciated, thus increasing their workplace anxiety.

Moreover, we argue that the positive effect of proactivity on work-family conflict through workplace anxiety may be weaker for employees with high EI. Specifically, we expect employee EI to weaken the indirect effects of proactivity on work-family conflict. Individuals with high EI are skilled at emotional expression, emotion identification, and emotion management. Therefore, they can handle negative affect and stress that may spill over from work to family (Edwards, 2006). Similarly, individuals with high EI are likely more adaptable in high-pressure conditions and are likely to see pressure as a challenge instead of as a threat (Schneider et al., 2013). Thus, good use of emotional skills might help people with high EI to have strong well-being and health (Zeidner et al., 2012; Sánchez-Álvarez et al., 2016). Based on the COR theory (Hobfoll, 1989) and the W-HR model (ten Brummelhuis and Bakker, 2012), EI is considered a key resource that might be useful in protecting against resource loss and aid in recovery from losses (Halbesleben et al., 2014). Therefore, employees with high EI may be better at coping with strain and anxiety prompted by proactivity, which may in turn reduce their work-family conflict caused by the resource loss. In contrast, proactive employees with low EI are unable to cope with the stress and anxiety caused by proactive behavior, which ultimately negatively affects their family lives. In summary, we hypothesize the following:

*Hypothesis 7*: EI moderates the positive relationship between proactive behavior and workplace anxiety so that the relationship is weaker for employees with high levels of EI (in contrast to employees with low levels of EI).

*Hypothesis 8*: EI moderates the positive impact of proactive behavior on work-family conflict *via* workplace anxiety so that the relationship is weaker for employees with high levels of EI (in contrast to employees with low levels of EI).

## Materials and Methods

### Instrument Development

In this study, a questionnaire was used for data collection (Rasool et al., 2019; Zhou et al., 2020). The questionnaire comprised 36 items (three items for proactive behavior, eight items for workplace anxiety, five items for work-family conflict, four items for immediate supervisor perspective taking, and 16 items for EI) scored with a five-point Likert scale (1 = strongly disagree and 5 = strongly agree). The Brislin (1980) back-translation procedure was employed for all measures, which were provided to participants in Chinese. To enhance the rigor of the study, a pilot study was conducted to check the instrument’s reliability and validity. An initial questionnaire was distributed to 32 professionals (12 professors, 13 PhD students, and seven human resource management specialists). These professionals recommended changes to the research instrument.

### Data Collection

To minimize common method bias, we used a two-wave, multisource (supervisor-subordinate dyads) design to collect data (Podsakoff et al., 2003). The data were collected for approximately 1 month from September to October 2020. We recruited seven human resource (HR) managers from banks, manufacturing companies, high-technology companies, and trading companies (one banking enterprise, three enterprises in the high-technology industry, two enterprises in the manufacturing industry, and one trading company) who attended a part-time MBA program in three cities (Changchun, Dalian, and Harbin) in Northeast China. After speaking with these HR managers about the survey procedure and content, we directed them to randomly recruit full-time employees in their organizations.

When the survey was conducted, we first communicated with the HR management department of each company, and the HR departments communicated and coordinated with all relevant departments and explained the purpose of our questionnaire survey. Each HR department was asked to provide the names and job numbers of volunteer supervisor-subordinate dyads. Survey questionnaires were matched by codes determined by the researchers. Two graduate psychology students who were independent from the organizations were assigned to conduct the surveys. The participants finished the questionnaire in their own time and returned it in a sealed envelope to the research assistant. The survey instructions informed the participants of the objectives of the study, and the confidential nature of the participants’ responses was strictly ensured. The participants were asked to record their identification numbers so that the individual responses could be matched over time. At time 1 (on a Friday afternoon), 130 supervisors of employees were invited to report their perspective taking and subordinate proactive behavior over the past week; 122 supervisors completed the survey (response rate of 93.84%). A total of 580 employees were invited to report their demographic information (gender, age, marital status, education, and tenure), EI, and workplace anxiety over the past week, and 494 completed the survey (response rate of 85.17%). After screening out unmatched records, 490 subordinates and 120 supervisors remained. At time 2 (the end of the weekend), we asked the subordinates to report the work-family conflict they experienced over the past weekend. The subordinates were asked to answer the questions between 1900 and 2,100 h. The researcher sent a message *via* WeChat to remind the subordinates to complete the questionnaire on time and asked them to take a picture of the questionnaire to give feedback. At time 2, 470 participants completed the survey. After excluding missing data and non-matched data, we ultimately obtained 450 valid questionnaires. To improve the quality of the questionnaire data, we provided compensation of approximately US $8 to the supervisors and US $5 to the subordinates.

### Measures

#### Proactive Behavior

The proactive behavior items were adopted from Griffin et al. (2007). The three items were rated on a five-point Likert scale (ranging from 1 = strongly disagree to 5 = strongly agree). A sample item is “I initiated better ways of performing my core tasks” (Cronbach’s *α* = 0.75; see Table 1).

**Table 1:** Results of the descriptive statistical analysis.

Variables	M	SD	1	2	3	4	5	6	7	8	9	10
1. Gender^a^	0.48	0.50										
2. Age^b^	3.27	1.18	0.00									
3. Marital status^c^	0.55	0.49	−0.05	0.12^**^								
4. Education^d^	2.64	0.87	−0.09^*^	0.00	0.13^**^							
5. Tenure^e^	2.79	1.42	−0.03	0.37^**^	0.18^**^	0.08						
6. Proactive behavior (time 1)	3.71	0.67	−0.11^*^	0.05	0.01	0.06	−0.09	(0.75)				
7. Workplace anxiety (time 1)	3.59	0.54	−0.12^**^	0.01	0.07	0.03	−0.02	0.58^**^	(0.88)			
8. Work-family conflict (time 2)	3.86	0.52	−0.10^*^	0.09^*^	0.10^*^	0.08	0.03	0.49^**^	0.57^**^	(0.83)		
9. Emotional intelligence (time 1)	3.39	0.95	−0.01	−0.02	−0.07	0.15^**^	0.22^**^	−0.03	−0.11^*^	−0.05	(0.95)	
10. Immediate supervisor perspective taking (time 1)	3.23	1.36	−0.00	−0.07	−0.04	−0.07	−0.04	0.06	0.03	0.03	−0.03	(0.89)

*n* = 450; Cronbach’s alpha reliability coefficients are displayed on the diagonal.

* *p* < 0.05;

** *p* < 0.01.

a Male = 0; Female = 1.

b Under 20 = 1; 20–25 = 2; 36–30 = 3; 31–35 = 4; 36–40 = 5; 41 and over = 6.

c Single = 0; Married = 1.

d High school = 1; College degree = 2; Bachelor’s degree = 3; Master’s degree and over = 4.

e Under 1 year = 1; 1–3 years = 2; 3–5 years = 3; 5–7 years = 4; 7 years and over = 5.

#### Workplace Anxiety

The workplace anxiety items were adopted from McCarthy et al. (2016). The eight items were rated on a five-point Likert scale (ranging from 1 = strongly disagree to 5 = strongly agree). A sample item is “Even when I try as hard as I can, I still worry about whether my job performance will be good enough” (Cronbach’s *α* = 0.88; see Table 1).

#### Work-Family Conflict

The work-family conflict items were adopted from Netemeyer et al. (1996). The five items were rated on a five-point Likert scale (ranging from 1 = strongly disagree to 5 = strongly agree). A sample item is “The demands of my work interfere with my home and family life” (Cronbach’s *α* = 0.83; see Table 1).

#### Immediate Supervisor Perspective Taking

The items on immediate supervisor perspective taking were adopted from Grant and Berry (2011). The four items were rated on a five-point Likert scale (ranging from 1 = strongly disagree to 5 = strongly agree). A sample item is “On the job, my supervisor will try to take my perspective” (Cronbach’s *α* = 0.89; see Table 1).

#### Emotional Intelligence

The EI items were adopted from Wong and Law (2002). The 16 items were rated on a five-point Likert scale (ranging from 1 = strongly disagree to 5 = strongly agree). Sample items include the following: “I always know my friends’ emotions from their behavior” (others’ emotion appraisals), “I have a good understanding of my own emotions” (self-emotion appraisal), “I always set goals for myself and then try my best to achieve them” (use of emotion), and “I am able to control my temper and handle difficulties rationally” (regulation of emotion; Cronbach’s *α* = 0.95; see Table 1).

#### Control Variables

Gender, age, marital status, education, and tenure have been identified as potential predictors of work-family conflict in previous studies (Bolino and Turnley, 2005; Byron, 2005; Gao et al., 2013). Following prior suggestions to use control variables (Carlson and Wu, 2012; Bernerth and Aguinis, 2016), we controlled for employees’ gender, age, marital status, education, and tenure to better estimate the effects of proactive behavior on work-family conflict.

### Demographic Information and Participant Summary

A total of 450 employees were randomly selected from seven enterprises from three cities (Changchun, Dalian, and Harbin) in Northeast China. Of the sample, 52.4% of the participants were male. Regarding their marital status, 55.3% were married. The age distribution of the sample was as follows: 18–20 years (0.9%), 21–25 years (28.7%), 26–30 years (35.1%), 31–35 years (19.1%), 36–40 years (10.2%), and 41 years or over (6%). A total of 48.9% of the participants had a bachelor’s degree, and approximately 48.7% had 1–5 years of work experience.

## Results

### Data Analysis Strategy

Firstly, statistical analyses were carried out with SPSS 24.0 to determine the reliability of the data and to calculate the descriptive statistics and correlations among the variables. Secondly, the measurement model was tested using confirmatory factor analysis (CFA) with AMOS 17.0 to assess the variables’ discriminant validity. Thirdly, the moderated mediation model was tested using PROCESS, as recommended by Muller et al. (2005). The mediating effect was assessed using 5,000 bootstrap estimates based on 95% bias-corrected confidence intervals (CIs; PROCESS, model 4; Hayes, 2013). The moderation model was tested using the PROCESS macro (model 7; Edwards and Lambert, 2007; Hayes, 2013). The CIs were calculated, and if they did not include zero, the null hypothesis was rejected in support of the study hypothesis.

### Descriptive Statistical Analysis

The means, SD, and correlations of the study variables are shown in Table 1. As expected, proactive behavior (time 1) was positively and significantly related to workplace anxiety (time 1; *r* = 0.58, *p* < 0.01) and work-family conflict (time 2; *r* = 0.49, *p* < 0.01). Additionally, workplace anxiety (time 1) was positively related to work-family conflict (time 2; *r* = 0.57, *p* < 0.01). Workplace anxiety (time 1) was also positively related to EI (time 1; *r* = −0.11, *p* < 0.05).

### Measurement Model

A CFA of the above five measures was conducted to analyze discriminant validity using AMOS 17.0 with maximum likelihood estimation procedures. As shown in Table 2, we found good support for the five-factor solution (proactive behavior, workplace anxiety, work-family conflict, immediate supervisor perspective taking, and EI), which showed an adequate fit to the data: *χ*^2^ = 1,493.81, degrees of freedom (*df*) = 568, comparative fit index (CFI) = 0.92, incremental fit index (IFI) = 0.92, Tucker-Lewis index (TLI) = 0.91, and root mean square error of approximation (RMSEA) = 0.060.

**Table 2:** Confirmatory factor analysis.

Measurement models	*χ*^2^	*df*	CFI	TLI	IFI	RMSEA
Five-factor model	1,493.81	568	0.92	0.91	0.92	0.060
Four-factor model (combining proactive behavior and workplace anxiety into one factor)	1,762.03	575	0.90	0.89	0.90	0.068
Three-factor model (combining proactive behavior, perspective taking, and EI into one factor)	2,861.31	578	0.82	0.81	0.83	0.090
Two-factor model (combining proactive behavior, perspective taking, work-family conflict, and EI into one factor)	4,882.19	579	0.63	0.60	0.63	0.132
One-factor model (combining all items into one factor)	5,623.46	580	0.59	0.55	0.59	0.139

CFI, comparative fit index; IFI, incremental fit index; TLI, Tucker-Lewis index; RMSEA, root mean square error of approximation.

### Hypothesis Testing

We used SPSS 24.0 to test our hypotheses. We followed the procedures recommended by Muller et al. (2005) and used multiple regression analysis to test our moderated mediation model. We centered proactive behavior, workplace anxiety, EI, and immediate supervisor perspective taking to reduce multicollinearity according to Aiken and West (1991).

Firstly, we tested the indirect effects. As shown in Table 3, the results from the regression analysis of the mediation model indicated that proactive behavior was positively and significantly associated with work-family conflict (model 7; *β* = 0.48, *p* < 0.001) and workplace anxiety (model 1; *β* = 0.58, *p* < 0.001). Workplace anxiety was positively associated with work-family conflict (model 8; *β* = 0.42, *p* < 0.001). Thus, Hypothesis 1, 2, and 3 were supported. To test the mediating effect, we assessed the mediation model using 5,000 bootstrap estimates based on 95% bias-corrected CIs (PROCESS, model 4; Hayes, 2013). The bootstrapping results showed that the indirect effect of proactive behavior on work-family conflict (indirect effect = 0.37, SE = 0.46, 95% CI = 0.29–0.47) was significant. Thus, Hypothesis 4 was supported.

**Table 3:** Moderated mediation analysis.

Variables	Workplace anxiety					Work-family conflict		
	Model 1	Model 2	Model 3	Model 4	Model 5	Model 6	Model 7	Model 8
Intercept	1.87^***^	1.89^***^	1.27^***^	2.05^***^	1.31^***^	3.65^***^	2.26^***^	1.48^***^
Age	−0.03	−0.03	−0.03	−0.05	−0.05	0.09	0.04	0.05
Gender	−0.05	−0.05	−0.05	−0.05	−0.05	−0.09^*^	−0.04	−0.01
Marital status	0.06	0.06	0.06	0.04	0.04	0.08	0.07	0.04
Education	−0.02	−0.02	−0.02	−0.00	0.01	0.06	0.03	0.04
Tenure	0.03	0.03	0.03	0.06	0.05	−0.02	0.04	0.03
Proactive behavior	0.58^***^	0.58^***^	0.79^***^	0.58^***^	0.83^***^		0.48^***^	0.23^***^
Workplace anxiety								0.42^***^
Immediate supervisor perspective taking		−0.01	0.48^*^					
Emotional intelligence				−0.010^*^	0.30			
Proactive behavior × Perspective taking			−0.56^**^					
Proactive behavior × Emotional intelligence					−0.49^**^			
*R*^2^	0.35	0.35	0.36	0.36	0.37	0.03	0.25	0.37
Adjusted *R*^2^	0.34	0.34	0.35	0.35	0.36	0.02	0.24	0.36
*F*	40.82^***^	34.94^***^	31.88^***^	36.57^***^	33.27^***^	2.86^*^	25.62^***^	38.00^***^

*n* = 450.

* *p* < 0.05;

** *p* < 0.01;

*** *p* < 0.001.

**Figure 2:**
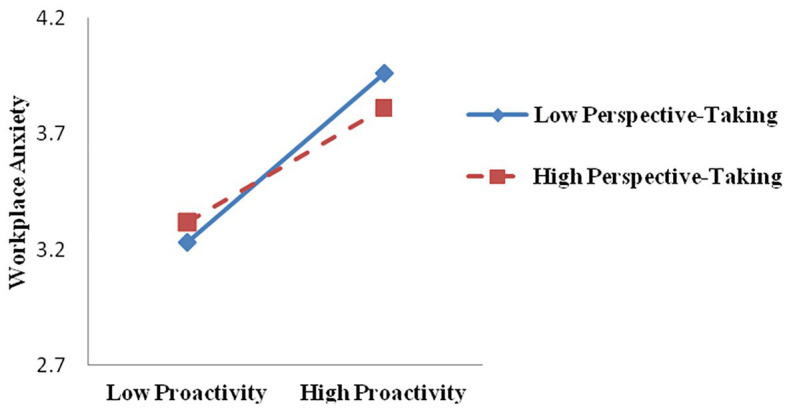
Moderating effect of immediate supervisor perspective taking on the relationship between proactive behavior and workplace anxiety.

Secondly, we tested the moderation model using the PROCESS macro (model 7; Edwards and Lambert, 2007; Hayes, 2013). In Hypothesis 5, we proposed the moderating role of immediate supervisor perspective taking in the positive relationship between proactive behavior and workplace anxiety. As shown in model 3 in Table 3, the interaction term of proactive behavior and immediate supervisor perspective taking was statistically significant (*β* = −0.56, *p* < 0.01). Furthermore, a simple slope test revealed that the effect of proactive behavior on workplace anxiety was stronger for low levels of immediate supervisor perspective taking (*β* = 0.54, SE = 0.03, *p* < 0.001) than for high levels (*β* = 0.39, SE = 0.04, *p* < 0.001; see Figure 2). Thus, Hypothesis 5 was supported. Moreover, we used the approach of Preacher et al. (2007) to test the conditional indirect effect. As indicated in Table 4, proactive behavior had a stronger positive and statistically significant conditional indirect effect on work-family conflict *via* workplace anxiety when the immediate supervisor had low levels of perspective taking (−1 SD, indirect effect = 0.22, SE = 0.35, 95% CI = 0.16–0.30) than when the immediate supervisor had high levels of perspective taking (+1 SD, indirect effect = 0.16, SE = 0.28, 95% CI = 0.11–0.22). Furthermore, the difference in the indirect effect was significant (*∆β* = −0.06, SE = 0.028, 95% CI = −0.12 to −0.00). Thus, Hypothesis 6 was supported.

**Table 4:** Conditional indirect effects.

Moderator	Level	Effect	Boot SE	Boot *p*	CI
Immediate supervisor perspective taking	Low (−1 SD)	0.229	0.354	0.000	0.165–0.305
	High (+1 SD)	0.116	0.280	0.000	0.116–0.226

*n* = 450. Bootstrapping repetitions, *N* = 5,000. CI, confidence interval.

**Figure 3:**
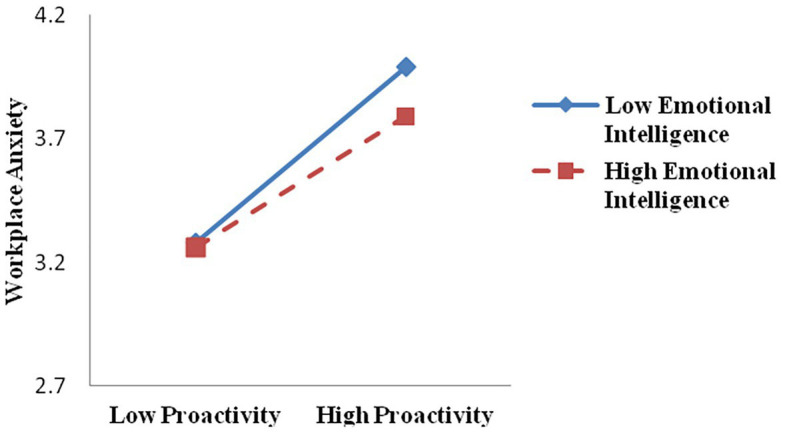
Moderating effect of employee emotional intelligence on the relationship between proactive behavior and workplace anxiety.

Thirdly, we also tested Hypothesis 7 and 8 using the PROCESS macro (model 7; Edwards and Lambert, 2007; Hayes, 2013). In Hypothesis 7, we proposed the moderating role of EI in the positive relationship between proactive behavior and workplace anxiety. As shown in model 5 in Table 3, the interaction term of proactive behavior and EI was statistically significant (*β* = −0.49, *p* < 0.01). Furthermore, a simple slope test revealed that the effect of proactivity on workplace anxiety was stronger for low EI (*β* = 0.51, SE = 0.03, *p* < 0.001) than for high EI (*β* = 0.39, SE = 0.04, *p* < 0.001; see Figure 3). Thus, Hypothesis 7 was supported.

Additionally, as indicated in Table 5, proactivity had a stronger positive and statistically significant conditional indirect effect on work-family conflict *via* EI when EI was low (−1 SD, indirect effect = 0.21, SE = 0.33, 95% CI = 0.15–0.28) than when EI was high (+1 SD, indirect effect = 0.16, SE = 0.27, 95% CI = 0.11–0.22). Furthermore, the difference in the indirect effect was significant (*∆β* = −0.05, SE = 0.027, 95% CI = −0.10 to −0.00). Thus, Hypothesis 8 was supported.

**Table 5:** Conditional indirect effects.

Moderator	Level	Effect	Boot SE	Boot *p*	CI
Emotional intelligence	Low (−1 SD)	0.218	0.336	0.000	0.157–0.228
	High (+1 SD)	0.116	0.270	0.000	0.114–0.222

*n* = 450. Bootstrapping repetitions, *N* = 5,000, CI, confidence interval

## Discussion

Drawing upon the COR theory (Hobfoll, 1989) and the W-HR model (ten Brummelhuis and Bakker, 2012), we developed and tested a moderated mediation model explaining how and when proactive behavior affects work-family conflict. In this study, we identified a positive theoretical link between proactive behavior and work-family conflict that strengthens the understanding of the outcomes of proactivity. Our empirical results support the proposition that proactivity is positively linked to workplace anxiety. We also found a mediating effect of workplace anxiety on the relationship between proactivity and work-family conflict and observed that immediate supervisor perspective taking can weaken the positive relationship between employee proactivity and workplace anxiety. Moreover, immediate supervisor perspective taking buffers the indirect effects of employee proactivity on employee work-family conflict such that the relationship is weaker (*versus* stronger) among employees whose supervisors demonstrate lower (*versus* higher) perspective taking. Our results also indicate that EI reduces the positive associations between proactive behavior and workplace anxiety. EI weakens the indirect effects of proactive behavior on work-family conflict such that the relationship is weaker (*versus* stronger) among employees with higher (*versus* lower) EI. Our findings contribute to the theory and research on proactivity and the work-family interface in three ways.

Firstly, our research shows that proactive behavior is positively associated with work-family conflict. To our knowledge, behavior-based conflict in the work-family conflict model by Greenhaus and Beutell (1985) has been relatively little examined in recent studies (Dierdorff and Ellington, 2008). Our results provide empirical support for the finding that proactive behavior spills over to the home domain and leads to work-family conflict. This result is consistent with those of previous studies and confirms the positive relationship between proactive behavior and work-family conflict (Bolino and Turnley, 2005; Harrison and Wagner, 2016; Zito et al., 2019). Furthermore, most previous studies have focused on the benefit of proactive behavior in organizations. Our results contribute to proactivity research by revealing the “dark side” of proactive behavior on the work-family interface. Our findings also answer the calls from Liu et al. (2019) and deepen our understanding of the consequences of proactive behavior.

Secondly, taking the COR theory and the W-HR model as our primary theoretical lens, we demonstrate through our research results that workplace anxiety plays a mediating role in the relationship between proactive behavior and work-family conflict. Although past studies have examined the relationship between specific proactive behavior and work-family conflict, investigation of the underlying mechanism of the relationship between proactive behavior and the work-family interface has been neglected (Bolino and Turnley, 2005; Harrison and Wagner, 2016; Zito et al., 2019). By examining the mediating effect of workplace anxiety, we provide empirical support for the idea that proactive behavior generates workplace anxiety, which in turn leads to work-family conflict. Hence, proactive behavior requiring resource consumption can lead to job strain and anxiety, which is more likely to spill over to the home domain and cause work-family conflict. Our results also extend and are consistent with the findings of Lin and Johnson (2015) and Pingel et al. (2019) that proactive behavior leads to resource loss, which results in anxiety and, ultimately, work-family conflict. Moreover, we found that proactive behavior is positively related to workplace anxiety; hence, behaving proactively has detrimental effects for employees. This result extends and highlights the findings by Cangiano et al. (2019) that proactive behavior was positively linked with workplace anxiety.

Thirdly, our results show that immediate supervisor perspective taking and employee EI moderate the indirect effects of proactive behavior on work-family conflict through workplace anxiety. While recent studies have argued that perspective taking can help leaders communicate and motivate subordinates more effectively (Galinsky and Schweitzer, 2016), few studies have empirically tested the role of leaders’ perspective taking in an organizational context. In line with the COR theory and the W-HR model, our study supports the idea that subordinates working under leaders with high levels of perspective taking can acquire more work resources, which in turn prevents resource loss from the work domain to the family domain. Moreover, our results show that employees’ EI can weaken the relationship between proactive behavior and work-family conflict. This result provides evidence that employees with high EI can regulate their emotions and consider the feelings of others at work, which in turn reduces their workplace anxiety caused by interpersonal tension from their proactive behavior. This finding is consistent with and extends the previous research of Amernic and Craig (2010). EI makes proactive employees pay more attention to social and relational contexts and consider their own self-regulation. Thus, employees who are proactive and have high EI seem to be “wiser,” which thus weakens the negative effects of proactive behavior. Meanwhile, our results confirm that EI can be considered a key resource in protecting against resource loss stemming from proactive behavior (Halbesleben et al., 2014).

## Conclusion

This study demonstrates that proactive behavior is positively related to work-family conflict and that workplace anxiety partially mediates this relationship. In addition, the study shows that immediate supervisor perspective taking and employee EI moderate the indirect effects of proactive behavior on work-family conflict through workplace anxiety. We provide empirical support for the idea that proactive behavior generates workplace anxiety, which in turn leads to work-family conflict. We hope that this study will spark interest among scholars in the darker side of proactive behavior. We also hope that practitioners will pay attention to the negative effects of proactive behavior on employees’ family lives while motivating them to take initiative.

## Practical Implications

Our findings highlight some significant managerial implications for organizations. Firstly, our results suggest that proactive behavior spills over to the home domain and proactively correlates with work-family conflict both directly and indirectly. Hence, it is vital for organizations to realize that employees’ proactive behavior may have detrimental effects on their family lives. Managers need to consider the “dark side” of proactive behavior and formulate supportive family policies for proactive employees. Organizations can also provide work-family segmentation policies, such as flextime, which can lower the impact of work on the family. These practices can help employees alleviate work-family conflicts caused by proactive behaviors. Secondly, our results show that proactive behavior is resource-consuming, which causes proactive individuals to experience workplace anxiety. Thus, organizations can formulate policies and provide resources for proactive employees; for example, organizational line managers should be encouraged to provide support such as flexible work options, job autonomy, and emotional support that conveys compassion and understanding to support proactive employees. Organizations can also create a more inclusive and compatible organizational culture to reduce workplace anxiety caused by proactivity. Thirdly, our study shows that immediate supervisor perspective taking is a boundary condition that benefits proactivity and related outcomes. This finding suggests that line managers’ perspective taking helps them to more easily and effectively communicate with and motivate proactive employees. Thus, organizations should select line managers who are highly agreeable and skilled at perspective taking and should provide training to supervisors to help them improve their perspective taking. Such training will promote understanding between line managers and proactive employees. This practice can also alleviate the anxiety that may be generated by proactive behavior. Lastly, given that EI moderated the relationship between proactive behavior and work-family conflict, organizations should pay more attention to the potential value of EI in human resource management practice. Specifically, organizations can make hiring decisions based on applicants’ EI and provide training courses to help employees improve their emotion management skills.

## Limitations and Future Research

Despite the findings and the contributions made to the field, this study had some limitations that should be addressed. Firstly, although we collected data with a multisource, time-lagged design, the potential threat of common method bias still cannot be excluded. Because work-family conflict may also affect workplace behavior, we encourage future research to use a longitudinal design to investigate the reciprocal relationship between proactive behavior and work-family conflict to clarify causal relationships. Secondly, our data were collected only from enterprises located in Northeast China. There is some question as to whether our findings can be replicated in other countries. Therefore, to improve the generalizability of the present findings, we encourage future research to use samples from Western countries to test the generalizability of the findings. Thirdly, our research was based on a resource theoretical perspective and focused only on the impact of proactivity on work-family conflict to construct a moderated mediation model. Since other potential factors may affect employees’ workplace anxiety and work-family conflict during the process, future research should identify other mechanisms between proactive behavior and work-family conflict. Finally, the work-family interface is generally considered culturally sensitive (Powell et al., 2009), and our research was conducted solely in the Chinese cultural context. Thus, a specific model that considers cultural characteristics (such as collectivism and power distance) as moderators might be more powerful in future studies.

## Data Availability Statement

The original contributions presented in the study are included in the article/supplementary material, further inquiries can be directed to the corresponding author.

## Ethics Statement

The studies involving human participants were reviewed and approved by Ethics Committee of Jilin University of Finance and Economics. The patients/participants provided their written informed consent to participate in this study.

## Author Contributions

ZC contributed to study conception theoretical foundation, model development, and research design. ZC and YL contributed to the literature research, analysis and interpretation of data, and drafting of manuscript. All authors contributed to the article and approved the submitted version.

### Conflict of Interest

The authors declare that the research was conducted in the absence of any commercial or financial relationships that could be construed as a potential conflict of interest.
